# HLA class I-drug-T-cell receptor interactions in SJS/TEN

**DOI:** 10.1186/2045-7022-4-S3-P2

**Published:** 2014-07-18

**Authors:** Craig Rive, Rebecca Pavlos, David Ostrov, Pablo Plasencia, Shien-Iu Hung, WenHung Chung, Bjoern Peters, Soren Buus, Simon Mallal, Elizabeth Phillips

**Affiliations:** 1 Institute for Immunology and Infectious Diseases, Murdoch University, Australia; 2 University of Florida, College of Medicine, USA; 3 Institute of Pharmacology, School of Medicine, Genome Research Centre, National Yang-Ming University, Taiwan; 4 Department of Dermatology, Chang Gung Memorial Hospital, College of Medicine, Chang Gung University, Taiwan; 5 La Jolla Institute for Allergy and Immunology, La Jolla, USA; 6 Laboratory of Experimental Immunology, University of Copenhagen, Denmark; 7 Institute for Immunology and Infectious Diseases, Murdoch University, Murdoch WA, Australia, and Vanderbilt University Medical Centre, USA

## Background

Carbamazepine (CBZ) is associated with the severe cutaneous drug reaction Stevens-Johnson Syndrome/toxic epidermal necrolysis (SJS/TEN). CBZ-SJS/TEN has been associated with HLA-B*15:02 carriage and specific T-cell clonotypes. We aimed to characterize the interactions between specific T-cell receptor (TCR) clonotypes, HLA-B*15:02 and CBZ.

## Methods

Peripheral blood mononuclear cells (PBMCs) were isolated from patients with CBZ-SJS/TEN and healthy controls, stimulated with 10ug/mL CBZ (Sigma, cat. no. C4024-5G) and cultured over a 9-14 day period. RNA was isolated at various time points and TCR V subtypes were assessed using digital droplet PCR (Bio-Rad QX100 droplet digital PCR system). Drug specific T-cell INF and granulysin responses were assessed by ELISpot and ICS. *In silico* modelling was used to examine the interaction between the known specific TCR V and V chains, CBZ and HLA-B*15:02.

## Results

CBZ specific T-cell INF and granulysin responses were detected many years following the original SJS/TEN reaction. Expansion of specific TCR CDR3 sequences was confirmed in CBZ SJS/TEN patient cultures. *In silico* modelling of the CBZ-HLA-B*15:02-TCR interaction suggests that CBZ binds non-covalently in the P4 binding pocket of the HLA-B*15:02 antigen binding cleft in a site that is typically occupied with bound peptide.

## Conclusions

These findings raise two non-mutually exclusive possibilities: 1) CBZ may block peptide binding and be presented by HLA-B*15:02 in a solvent exposed manner available for direct recognition by the TCR, 2) CBZ binding the central portion of the HLA-B*15:02 antigen binding cleft may permit long peptides to bind conventionally at the peptide termini (in the A and F pockets) and bulge over the drug in the central residues to permit indirect recognition of CBZ (peptide mediated TCR contact).

